# Adaptation and testing of an assessment for mental health and alcohol use problems among conflict-affected adults in Ukraine

**DOI:** 10.1186/s13031-018-0169-6

**Published:** 2018-08-15

**Authors:** Benjamin Doty, Emily E. Haroz, Namrita S. Singh, Sergiy Bogdanov, Judith K. Bass, Laura K. Murray, Karis L. Callaway, Paul A. Bolton

**Affiliations:** 1 Department of Mental Health, Johns Hopkins Bloomberg School of Public Health, Baltimore, MD USA; 2 Department of International Health, Johns Hopkins Bloomberg School of Public Health, Baltimore, MD USA; 3 Center for Mental Health and Psychosocial Support, National University of Kyiv-Mohyla Academy, Kyiv, Ukraine; 4 Department of Psychology, Western Michigan University, Kalamazoo, MI USA

**Keywords:** Validity, Reliability, Internally displaced, Veteran, Ukraine, Depression, Post-traumatic stress, Anxiety, Alcohol

## Abstract

**Background:**

In Ukraine, a large number of internally displaced persons (IDPs) and veterans experience social and psychological problems as a result of the ongoing conflict between Ukraine and Russia. Our purpose was to develop reliable and valid instruments to screen for common mental health and alcohol use problems in these populations.

**Methods:**

We used a three-step process of instrument adaptation and testing. The instrument—the Mental Health Assessment Inventory (MHAI)—combines adapted standard screeners with items derived locally in Ukraine. A validity study was conducted using a sample of 153 adults (54% male) ages 18 years and older. All participants in the sample were IDPs or veterans living in or near the major urban areas of Kyiv and Zaporizhia. Reliability testing (internal consistency, test-retest) and validity testing (construct, criterion) of the MHAI were conducted using classical test theory. After initial testing, we used Item Response Theory (IRT) to shorten and further refine the instrument.

**Results:**

The MHAI showed good internal consistency and test-retest reliability for the main outcomes: depression (**α** = 0.94; *r* = .84), post-traumatic stress (PTS; **α** = 0.97; *r* = 0.87), anxiety (**α** = 0.90; *r* = 0.80), and alcohol use (**α** = 0.86; *r* = 0.91). There was good evidence of convergent construct validity among the scales for depression, PTS, and anxiety, but not for alcohol use. Item Response Theory (IRT) analysis supported use of shortened versions of the scales for depression, PTS, and anxiety, as they retained comparable psychometric properties to the full scales of the MHAI.

**Conclusion:**

The findings support the reliability and validity of the assessment—the MHAI—for screening of common mental health problems among Ukrainian IDPs and veterans. Use of IRT shortened the instrument to improve practicality and potential sustainability.

**Electronic supplementary material:**

The online version of this article (10.1186/s13031-018-0169-6) contains supplementary material, which is available to authorized users.

## Background

In late 2013 and early 2014, Ukraine underwent a period of rapidly escalating and widespread political discontent, resulting in the Ukrainian revolution of 2014 (also known as the Euromaidan Revolution) [1] and a prolonged, ongoing conflict in the eastern regions of the country [2, 3]. Since the conflict began, more than 10,000 people have been killed and more than 25,000 people have been wounded, including many veterans of the “Anti-Terrorist Operation” (ATO), the Ukrainian government’s military effort to defeat pro-Russian separatists from eastern Ukraine [4]. The conflict has severely disrupted social and economic life in eastern Ukraine, particularly in the oblasts (i.e., administrative regions) of Donetsk and Luhansk (the Donbass). More than 1.7 million Ukrainians—4% of the total population—have been internally displaced [5]. Reviews of mental health problems among diverse conflict-affected populations, including internally displaced persons (IDPs) and military veterans, reveal that displacement and exposure to violence are consistently associated with elevated psychiatric symptomatology, especially related to depressive, anxiety, and stress-related disorders [6, 7].

Beginning in 2015, the United States Agency for International Development’s (USAID) Victims of Torture Program began supporting mental health and psychosocial research and program activities to increase access to effective and appropriate services for the conflict-affected population in Ukraine. Johns Hopkins University (JHU) and its primary implementation partner, the National University of Kyiv-Mohyla Academy (NaUKMA), were chosen to lead this effort, which includes the identification and treatment of mental health problems among IDPs, veterans, and others affected by the conflict.

To enhance local capacity to identify conflict-affected individuals with mental health problems, we aimed to develop a set of reliable and valid instruments for self-report of common mental health problems among adult Ukrainian IDPs and ATO veterans. Although some structured assessments of mental health problems have been used in Ukraine (e.g., Composite International Diagnostic Interview), [8, 9] to our knowledge there has been no local validation of self-report measures for these problems in any Ukrainian population. The validity study described herein entailed a process of instrument adaptation and testing and was part of a methodology for the design, implementation, monitoring, and evaluation (DIME) of community-based services to address mental health needs [10]. In addition to the standard DIME process, we utilized Item Response Theory (IRT) methods to shorten and refine the instruments to make them more pragmatic for use in both research and clinical practice.

## Methods

### Design

We used a three-step process: 1) instrument adaptation and translation; 2) instrument reliability and validity testing; and 3) instrument refinement. The adaptation process was designed to produce a draft set of measures attentive to the local culture and context [10]. Instrument testing consisted of piloting and data analyses to evaluate reliability and validity based on classical test theory [11]. Item Response Theory (IRT) methods and clinical feedback resulted in shortened scales that demonstrate comparable psychometric properties to the full scales.

#### Instrument adaptation and translation

The assessment instrument was developed by combining existing measures of common mental health problems and functioning with items drawn from a rapid qualitative study (conducted February–March 2016) among Ukrainian IDPs, Ukrainian military veterans of the ATO, and people familiar with the mental health problems of IDPs and veterans, including formal service providers. The qualitative study (see Singh et al., forthcoming) investigated local perspective on the psychosocial problems and challenges facing IDPs and veterans affected by the armed conflict between Ukraine and Russia. Depression, anxiety, trauma, and substance use emerged as the salient psychosocial problems. We searched the research literature for previously validated screening tools for these problems in Ukraine or similar populations (populations affected by conflict/community violence in eastern Europe or the former Soviet Union). We searched PubMed and Google Scholar, and the Russian and Ukrainian language literature as well. We found published studies of mental health research in Ukraine using structured interviews (e.g., Composite International Diagnostic Interview) [8, 9, 12], but no studies that tested the local validity of self-report measures for common mental health problems in adults.

Lacking locally validated measures, we tried to match the findings of the qualitative study to instruments our team had previously developed for cross-cultural use: the International Depression Symptoms Scale (IDSS) [13] and the Global Post Traumatic Stress Symptom Scale (GPTSS) [14]. The IDSS and GPTSS are self-report measures made up of a core set of symptoms—for depression and post-traumatic stress, respectively—that have been found through literature review to occur across diverse populations. They can therefore form a starting point for instrument adaptation and testing in the absence of proven alternatives. The IDSS includes 27 core symptoms, one item assessing functional impairment, and one item assessing suicidal ideation. The GPTSS includes 47 core symptoms: 32 symptoms specific to GPTSS and 15 symptoms overlapping with the IDSS. We found a high degree of overlap, with nearly all depressive and trauma-related symptoms mentioned in the qualitative findings represented in either the IDSS or GPTSS. An additional item identified during the qualitative study was added to the IDSS measure (a depressive symptom about ‘avoiding others,’ избегание других [Russian], izbeganie drugih [transliterated]).

Since anxiety and substance use were identified as important problems during the qualitative study, we included measures of both. We were unable to find self-report measures previously used in Ukraine, so we selected the 10-item anxiety sub-scale of the Hopkins Symptom Checklist (HSCL-A) [15, 16] given its demonstrated validity in diverse populations including those affected by political conflict [17–20]. For the IDSS, GPTSS, and HSCL-A measures, respondents were asked to report on the frequency they experienced each symptom in the previous two weeks. Response options included “none of the time” (0), “a little of the time” (1), “most of the time” (2), and “all of the time” (3). Average scale scores for depression, post-traumatic stress, and anxiety were generated separately for each participant by taking the mean of the responses on each scale.

We used the third version of the Alcohol Smoking and Substance Involvement Screening Test (ASSIST 3.0) to measure substance use [21]. The ASSIST 3.0 includes seven questions in each of 10 substance categories (alcohol, tobacco, cannabis, cocaine, amphetamine type stimulants, inhalants, sedatives, hallucinogens, opioids, and other). Questions focus on symptoms of dependence, frequency of use, substance-related functional impairment, and health and social consequences of use, usually over the previous three months. We explored alcohol use only, as this was by far the most frequently mentioned substance in the qualitative study. We scored responses according to recommended guidelines [21].

We used two assessments of functioning: the 12-item self-administered version of the World Health Organization’s Disability Adjustment Schedule 2.0 (WHODAS 2.0) [22] and a set of locally relevant function items that assesses frequent tasks and activities. The 12-item WHODAS 2.0 was developed to provide a standardized measurement of functional impairment across cultures. For each activity, participants report on their difficulty doing the activity because of health conditions. Example items include “standing for long periods of time, such as 30 minutes” and “taking care of household responsibilities.” The recommended 5-point ordinal response scale was used (0 = “none” [no difficulty] to 4 = “extreme [difficulty] or could not do”). We scored responses as a sum of item totals, according to recommended guidelines [22].

The set of locally relevant function items was developed from the qualitative study using methods described elsewhere [23, 24] in order to assess tasks and activities salient to the local context. We identified 21 items that cover tasks and activities that people regularly do to care for themselves, to care for their family, and to contribute to their community (e.g., “taking care of your personal appearance”). Participants reported the difficulty they had doing each task or activity in the past 30 days compared to other people of the same sex and age. A 5-point ordinal response scale was used: “no more [difficulty than others]” (0), “a little bit more” (1), “a moderate amount more” (2), “a lot more” (3), and “so much more difficulty that I often cannot do the activity” (4). A sixth response option, “not applicable,” was available for questions that were not relevant (e.g., caring for children if the respondent had no children). An average item score was generated for each study participant by calculating the mean score of all applicable items.

The study questionnaire included demographic questions (age, sex, marital status, education level, and current host community/study site [Kyiv or Zaporizhia]) as well as a checklist of 21 locally relevant traumatic events including items based on the qualitative study data (e.g., combat experience, assault with a weapon, sexual assault, forced displacement, and sudden loss of possessions to the point of poverty) [25]. For each of the 21 events, participants were asked to report their lifetime exposure: “learned about it happening to a close family member or friend” (1), “witnessed it happen to someone else” (2), and/or “happened to me personally” (3). Respondents could mark more than one option for each traumatic event. If the respondent had not experienced an event, they could indicate “not applicable to me” (0).

For convenience we refer to this set of instruments collectively as the Mental Health Assessment Inventory (MHAI; the instrument is provided in Additional file 1: Appendix A). A Ukrainian clinical psychologist (author SB) and two masters-level assistants, fluent in English, Russian, and Ukrainian, translated the MHAI into Russian. The MHAI was then back-translated into English to verify accuracy of translation. Local items added based on the qualitative data were phrased based on the wording provided by the interviewees.

The instruments were in Russian because most IDPs were from Russian-speaking regions. Item translations were checked against the qualitative data to ensure local phrasings and wordings were used whenever possible. The translated instrument was presented to study interviewers during their training session (led by authors BD, NS, and SB). All interviewers held a graduate degree in psychology or psychiatry. Interviewers reviewed the instrument instructions and items for linguistic and conceptual clarity. Potentially problematic items were noted and re-checked by the translation team. Russian versions of the WHODAS 2.0, ASSIST 3.0, and the Structured Clinical Interview for DSM-IV-Research Version (see below) were already available.

#### Instrument reliability and validity testing

The Structured Clinical Interview for DSM-IV-Research Version (SCID-IV-RV, 2010 revision) [26] was used as the study’s validation criterion. The SCID was developed for evaluation of DSM-defined disorders and places an emphasis on diagnostic simplicity and flexibility for adaptation to the study context/population. It is highly customizable, allowing researchers to select the modules/components that are relevant to a particular study. Items can also be rephrased as well as dropped from or added to the standard SCID modules to improve case identification [27–31].

We used the modules for mood episodes (Module A), mood disorders (Module D), substance use disorders (Module E; alcohol use symptoms), anxiety disorders (Module F), and current adjustment disorder (Module I). We slightly modified (see below) the SCID criteria for Major Depressive Disorder (MDD) and Post-traumatic Stress Disorder (PTSD) to improve the likelihood of capturing probable cases. We made these modifications after interviewers reported a pattern of participants not receiving a SCID diagnosis despite endorsing severe symptoms/functional impairment (i.e., the participants had severe depressive and/or post-traumatic stress symptoms but a subthreshold number of them to receive a diagnosis). On the interviewers’ clinical judgment, these clients were still being referred to mental health services. To reduce the number of false-negatives [32, 33] we followed the approach of other researchers [34] and broadened the SCID inclusion criteria—in this case, to include clients with some symptoms of MDD and PTSD but who were functionally impaired or needed treatment (i.e., the interviewer determined the participant’s condition was sufficiently severe to warrant referral to mental health services). The flexibility of semi-structured interviews for these sorts of modifications is an important advantage of their use, especially in mental health research in different cultural contexts.

Standard SCID diagnosis of MDD is based on the presence of five or more symptoms plus functional impairment. We modified the MDD diagnosis to be based on the presence of 4 or more symptoms plus functional impairment *or* recommendation for treatment services. Standard diagnosis of PTSD is based on meeting SCID criteria A (exposure to a traumatic event), B (re-experiencing the event), C (persistent avoidance), D (arousal), E (symptom duration), and F (functional impairment). We modified the PTSD diagnosis to be based on combinations of these criteria (see below) and recommendation for services. A participant was diagnosed with PTSD if s/he met one of three conditions:

1. 2+ of criteria A, B, C, D plus either criterion F or recommendation for services;
2. 3+ of criteria A, B, C, D plus criterion E; or.
3. all of criteria A, B, C, and D.

We used standard SCID criteria to diagnose Alcohol Abuse (criterion A) and Dependence (three dependence items coded 3). As only two participants met SCID diagnostic criteria (A-F) for Generalized Anxiety Disorder, we could not assess criterion validity for the HSCL anxiety sub-scale.

Data collection took place in the urban areas of Kyiv and Zaporizhia from June to August 2016. Kyiv and Zaporizhia host large numbers of IDPs. The study team engaged representatives from non-governmental organizations and community-based organizations in both sites to assist with sample recruitment by referring their clients to the study. In making their referrals, recruiters were asked to create two lists of potential study participants: one list of individuals with (one or more) mental health problems and one list of individuals the recruiter was confident did not have any mental health problem. Each recruiter was instructed how to identify individuals with symptoms of depression, post-traumatic stress, and/or alcohol misuse. Recruiters were provided with an information sheet containing brief descriptions of these problems along with inclusion criteria for the study. Potential participants were read a brief description of the study, and if they expressed interest in participating, they were also asked if they would be willing to be contacted by the research team. Recruiters documented names and phone numbers of interested individuals and transferred this information to the study coordinators. As referrals were made, the study coordinators contacted the potential participants by phone and read a recruitment script explaining the purpose of the study. If the participant agreed to meet in person to learn more about the study, a meeting time and place was set up by the coordinator. All participants provided in-person informed consent to the study interviewer on the day of the interview. Interviews took place in the participant’s home, the interviewer’s usual clinic, or in the offices of our local research partner, the NaUKMA Center for Mental Health and Psychosocial Support.

Our target population was people exposed to conflict (violence) or displacement due to the war in eastern Ukraine and Crimea. Outside of the Donbass, this population largely comprises IDPs and military veterans of the ATO. Inclusion criteria included: being age 18 and over and either an IDP or ATO veteran. Exclusion criteria included: being a danger to oneself (i.e., suicidal) or others; active psychosis; and/or having a major developmental delay. Study interviewers were trained mental health professionals who used their clinical judgment to assess whether exclusion criteria were met. An individual was excluded if s/he expressed symptoms or observable signs to the interviewer of active psychosis, major developmental delay, or intent to harm oneself or others.

##### Interviewer training

In early May 2016, we conducted an eight-day in-person training in Kyiv with the study interviewers (*n* = 8). All interviewers were Ukrainian citizens currently working in Ukraine as mental health professionals. The purpose of this training was to provide training and practice in the SCID. Interviewers received three days of didactic lectures (by authors SB and KC), video tutorials, group discussions, and role play exercises. The interviewers then conducted one to three practice SCID evaluations with 20 IDP interviewees who had agreed to be interviewed and video-recorded for the interviewers’ training purposes. De-identified SCID practice information was monitored to provide feedback to each interviewer to improve consistency and reliability of SCID administrations. All practice SCID administrations had at least one reviewer, different from the interviewer, simultaneously scoring and observing the administration. Practice continued until reliability between each interviewer and one of three gold standard raters (author SB, author KC, and a consultant hired from Zaporizhia) exceeded 90%.

In late May 2016, we conducted a second two-day interviewer training in Kyiv on study procedures and administration of the MHAI using tablet-based self-report. The training included a review of the study design and measurement tools, as well as research ethics principles, obtaining informed consent, assessing risk, responding to suicide ideation, practical exercises on proper data collection, familiarization with the study protocol, and practice and role-plays using the tablets and mobile data collection platform (Magpi) [35]. In June we carried out additional practice interviews and reliability testing of SCID interviewers in Kyiv and Zaporizhia to improve inter-rater reliability scores.

##### *Interview procedure*

All participants provided oral consent. During consent, participants were told the purpose of the study was to test mental health screening instruments to help local organizations correctly identify individuals in need of mental health services and to assist in measuring/tracking symptoms over time during/after receiving such services. After providing informed consent, participants completed a self-administered electronic version of the MHAI on a tablet computer using Magpi survey software [35]. The interviewer remained in the room with the respondent but sat on the opposite side of the room to be available to help operate the tablet or answer questions as needed while otherwise providing privacy. After a 15-min break, the interviewer then administered the SCID evaluation during which the interviewer recorded the participants’ responses on a paper version of the SCID; the interviewer later uploaded the responses to Magpi. To administer the SCID to the participants, interviewers used the SCID administration guidelines on which they had been previously trained. SCID trainers were available to consult with the interviewers by phone if needed.

On average, the MHAI took approximately 75 min to complete, and the SCID took 1 h. A random sample of participants was asked to return within 1 week for a repeat tablet-based administration of the MHAI by the same interviewer, for test-retest reliability analyses. Thirty of the 153 initial participants were re-interviewed (the re-interview sample) within 1 week (mean = 4 days; SD = 2 days) of the initial interview. Participants were compensated with a small gift (value of less than $10.00 USD; e.g., meal voucher) and, if applicable, a reimbursement for travel expenses.

##### *Ethical approval*

All study procedures were approved by the Institutional Review Boards of the Johns Hopkins Bloomberg School of Public Health in Baltimore, Maryland, USA (Protocol #6994) and the National University of Kyiv-Mohyla Academy in Kyiv, Ukraine (Protocol #02/559).

##### *Data analysis*

Data analysis incorporated standard evaluations of reliability (i.e., internal consistency, test-retest) and validity (i.e., criterion, construct). The estimated sample size needed for these analyses (*n* = 135) was based on the intended criterion validity analyses for the primary outcomes: depression and post-traumatic stress. Based on a two-tailed *t*-test for the difference between two independent groups (cases vs. non-cases according to the SCID) and an ability to detect an effect size of d = 0.60, with 80% power and a 0.05 type I error rate, we sought to recruit at least 45 participants meeting SCID criteria for MDD, 45 meeting SCID criteria for PTSD, and 45 not meeting criteria for either condition.

Reliability testing of the individual MHAI scales (depression, post-traumatic stress, anxiety, alcohol use, and functional impairment) included evaluation of test-retest reliability and internal consistency reliability. For test-retest analyses, 30 of the 153 participants were randomly selected to participate in a repeat interview. Based on prior work by ourselves and other researchers in other conflict-affected low- and middle-income populations, 30 is sufficient for test-retest analyses [36, 37]. To account for non-normal distribution of scale scores, test-retest reliability was calculated using Spearman’s rank order correlation coefficients (ρ). Internal consistency reliability was evaluated using Cronbach’s alpha (α) scores for each scale for both the initial and re-interview samples. Cronbach’s α and Spearman’s ρ both range from 0 to 1, with higher values indicating stronger correlations. Coefficients above 0.70, 0.80 and 0.90 are considered adequate, good, and strong, respectively [11, 38].

Convergent construct validity was assessed for all MHAI scales by assessing the degree to which each symptom scale correlated (Pearson’s [r] or Spearman’s [ρ] correlation coefficients) with one another and with other relevant scales. Although there is no cut-off that defines construct validity, larger coefficients are evidence of better validity. Correlations above 0.30 and 0.50 are generally considered moderate and strong, respectively. We anticipated the symptom scale scores would correlate positively with one another, whereas we anticipated the scale scores would inversely correlate with functioning. We used multiple indicators of functioning: the local functioning scale; the first 12 items of the WHODAS 2.0; and the single item on the IDSS about difficulty doing usual tasks at work/home.

Criterion validity was evaluated for the MHAI depression, post-traumatic stress, and alcohol use scales by comparing scores with SCID-IV-RV modified diagnoses for MDD and PTSD, as well as for Alcohol Abuse and Alcohol Dependence. T-tests were used to examine group differences between SCID-defined cases and non-cases. Receiver-operator characteristic curves and associated area under the curve (AUC) statistics were generated to examine how well the MHAI scales were able to distinguish between cases and non-cases. In the evaluation of Alcohol Abuse and Alcohol Dependence, we excluded participants who stated they had never used alcohol. An AUC of 1 means the test perfectly differentiates between case and non-case, whereas an AUC of 0.5 indicates differentiation no better than by chance alone, and an AUC of 0 means the test incorrectly classified all cases and non-cases. Empirical cut-points and associated sensitivity and specificity values were generated using the Liu method [39].

#### Instrument refinement using item response theory

Following completion of the reliability and validity testing, we used Item Response Theory (IRT) methods to produce a shortened assessment tool that would still reliably and validly identify individuals with mild, moderate, and severe depression, post-traumatic stress, and alcohol use problems with similar precision to the full MHAI instrument. IRT is a type of latent variable analysis that models the probability of endorsing individual items based on the level of the latent trait (e.g., severity of depression). IRT analyses have been used extensively in educational testing situations and more recently for questionnaire development and refinement of health outcome measures. Use of IRT can help in investigating systematic response bias as well as in refining and shortening instruments to reduce respondent burden [40].

Prior to conducting the IRT analysis, we used principal components analysis (PCA) to examine underlying dimensionality of the data and inform subsequent specification of IRT models. Graded Response Models (GRMs) [41] were performed for each scale on the MHAI as the most appropriate model given the ordered response categories of the items. In a GRM, both item discrimination (*a*) and item location (*b*) parameters are estimated for each item. Discrimination parameters are the same as factor loadings, indicating how strong an item is related to the underlying trait and how well it discriminates between different levels of this latent trait. Values of 0.01–0.34 are considered very low; 0.35–0.64 low; 0.65–1.34 moderate; 1.35–1.69 high; and 1.70 and above, very high [42]. Location parameters (or difficulty parameters) represent the level of the underlying latent trait where the probability of endorsing a particular item with a particular response category is 50%. In GRMs, multiple location parameters corresponding to each item response category are estimated (*b*_*1*_, *b*_*2*_*, b*_*3*_). The first location parameter (*b*_*1*_) represents the level of the underlying latent trait where the probability of endorsing the item with a “0” instead of a “1,” “2,” or “3” is 0.50; *b*_*2*_ is for the response of < 2; and *b*_*3*_ for the response of < 3. For the function items, due to an additional response category (0–4 instead of 0–3), four location parameters were estimated.

The criteria we used to select items for the shortened scales included: 1) high discrimination; 2) location parameters that represented a wide range of the latent trait; 3) adequate reliability of item responses in the original analysis; and 4) consideration of salience to the local population (based on previous qualitative study) or clinical utility. We first selected items based on criteria 1–3 and then added additional items based on criterion 4.

We created three short scales that measured MDD, PTS, and anxiety. Once items were selected for the shortened scales, we re-ran the reliability and validity analyses using the same procedure as previously described, including examining internal consistency reliability, test-retest reliability, convergent construct validity, and concurrent criterion validity. We compared these results to the longer scales. In addition, we examined reliability of the measure (or “information”) for the scale as a whole, using test information curves (TIC). Test information curves model the reliability of the scale as a function of the underlying latent trait allowing us to examine precision of the scales over the range of symptoms severity. We examined the TICs to ensure that the short scales were performing with comparative reliability to the long scales over the range of the latent trait.

All analyses were conducted using Stata statistical software, version 14 [43].

## Results

### Participant characteristics

A summary of participants’ demographic characteristics is provided in Table 1. In total, 153 participants (109 in Zaporizhia, 44 in Kyiv) were interviewed. The sample included adult IDPs (55%) and veterans. The status of five (3%) participants is unknown. There were slightly more male than female participants (54% vs. 46%). The majority were married (56%) or single (20%). Overall, the sample was highly educated; over half of participants (58%) had received at least a university degree. There were no statistically significant demographic differences between the re-interview sample and the single interview sample.

**Table 1:** Sample characteristics

	Baseline	
	*n* = 153	
	n	%
Mean age in years (SD)	39 (11)	
Site		
Kyiv	44	29
Zaporizhia	109	71
Male	83	54
Marital status		
Single	31	20
Married	86	56
Widowed	9	6
Divorced	27	18
Education		
Primary	4	3
High School	18	12
Vocational	42	27
University	83	54
Post-university	6	4
Status		
IDP	84	55
Veteran	64	42
Non-disclosed	5	3

Participants’ reports of exposure to traumatic events are shown in Table 2. Overall, we found high levels of exposure to traumatic events in our sample. Most participants had *experienced* combat exposure (84%). Other common exposures were lost contact with loved ones/fearing for their safety (58%), physical assault (46%), and forced displacement (46%). Many participants also reported witnessing life-threatening illness/injury (44%).

**Table 2:** Frequencies and percentages of participants’ reports of lifetime exposure to potentially traumatic events

	Experienced		Witnessed		Heard About	
Type of event*	n	%	n	%	n	%
Combat or exposure to war zone	128	84	57	37	26	17
Lost contact with loved ones and fear for their safety	88	58	10	7	23	15
Physical assault	71	46	30	20	21	14
Forced displacement	71	46	38	25	29	19
Fire or explosion	62	41	5	3	19	12
Sudden loss of possessions to the point of poverty	61	40	4	3	13	9
Transportation accident	53	35	35	23	9	6
Assault with a weapon	52	34	21	14	48	32
Severe human suffering	48	32	30	20	28	18
Life-threatening illness/injury	43	28	67	44	31	20
Starvation or fear of starvation	42	27	49	32	45	29
Serious accident during work/home/recreational activity	21	14	36	23	58	38
Other unwanted/uncomfortable sexual experience	17	11	10	7	7	5
Exposure to toxic substance	16	10	15	10	10	7
Serious injury, harm, or death you caused to someone else	16	10	19	12	11	7
Sexual assault	15	10	25	16	20	13
Natural disaster	13	9	36	24	17	11
Captivity	3	2	28	18	10	7
Sudden, violent death	–	–	12	8	34	22
Sudden accidental death	–	–	68	44	17	11
Any other very stressful event/experience	105	69	41	27	32	21

^*^The sample size for each response option is 153. Multiple responses were possible for each item; row percentages may not sum to 100. Column values reflect the number/percentage of respondents who responded "yes" to having experienced/witness/heard about the item

### Reliability of the MHAI instrument

Table 3 presents the results for internal consistency and test-retest reliability for the full MHAI scales and the shortened scales based on the IRT analysis. All Cronbach’s alpha (α) values for the full and shortened scales were acceptable, as evidenced by scores greater than α = 0.70. On the full MHAI scales, there were minor if any differences between the baseline and re-interview samples for PTS (α = 0.97 vs. 0.97), anxiety (α = 0.90 vs. 0.89), depression (α = 0.94 vs. 0.93), and alcohol use (α = 0.86 vs. 0.87) The baseline sample had somewhat higher alpha scores than the re-interview sample for the local functioning scale (α = 0.92 vs. 0.78), and the WHODAS scale (α = 0.95 vs. 0.80). Results were similar for long and short scales for depression (α = 0.94 vs. 0.89), PTS (α = 0.97 vs. 0.91), and anxiety (α = 0.90 vs. 0.82).

**Table 3:** Reliability results of mental health symptom and functioning scales

	Int’l depression symptom scale (IDSS)	Global post-traumatic stress scale (GPTSS)	HSCL anxiety sub-scale	ASSIST 3.0 alcohol scale	Local functioning scale	WHODAS 2.0 functioning scale
Internal consistency (α)						
Baseline sample (*n* = 153)	0.94	0.97	0.90	0.86	0.92	0.95
Re-interview sample (*n* = 30)	0.93	0.97	0.89	0.87	0.78	0.80
IRT-based analysis						
Shortened scale	0.89	0.91	0.82	–	–	–
Test-retest (ρ)						
Re-interview sample	0.84	0.87	0.80	0.91	0.85	0.90
IRT-based analysis						
Shortened scale	0.87	0.87	0.80	–	–	–

Test-retest reliability scores of the full MHAI scales for post-traumatic stress (*r* = 0.87), depression (*r* = 0.84), and anxiety (*r* = 0.80) were good, while reliability for alcohol use (*r* = 0.91), as measured on the ASSIST 3.0, was excellent. Test-retest reliability for functioning was excellent for the WHODAS (*r* = 0.90) and moderately high for the local functioning scale (*r* = 0.85 ). Nearly identical test-retest reliabilities were produced in the short scales for depression (r = 0.84 vs. 0.87), traumatic stress (r = 0.87 vs. 0.87), and anxiety (*r* = 0.81 and 0.80).

### Convergent validity

For the full MHAI scales, we observed a very high correlation between depression and PTS (*r* = 0.94) as well as high correlations between depression and anxiety (r = 0.84) and between anxiety and PTS (*r* = 0.79). The correlations between alcohol use and the mental health problem scales were low (depression: *r* = 0.18; PTS: *r* = 0.25; anxiety: *r* = 0.11). For the short scales resulting from the IRT analysis, we also observed a very high correlation between depression and PTS (r = 0.94). The correlation was acceptable between PTS and anxiety (*r* = 0.70) and moderate between depression and anxiety (*r* = 0.67). Compared to the full scales, the short scales between alcohol use and the mental health problems scales improved (depression: *r* = 0.31; PTS: *r* = 0.28; anxiety: *r* = 0.33).

For the full MHAI functioning scales, we found moderate-to-strong correlations between the WHODAS and depression (*r* = 0.69), PTS (r = 0.70), and anxiety (*r* = 0.51). The correlation between the WHODAS and alcohol use scale was low (*r* = 0.18). The local functioning scale correlations with depression (*r* = 0.71), post-traumatic stress (*r* = 0.76), anxiety (*r* = 0.52), and alcohol use (r = 0.18) were similar to the correlations between these and the WHODAS. For the short scales resulting from the IRT analysis, we found moderate-to-high correlations between the WHODAS and depression (*r* = 0.66), PTS (r = 0.69), and anxiety (r = 0.51). We also found relatively strong correlations between the local functioning scale and depression (0.78), PTS (0.77), and anxiety (0.69).

In the full MHAI, we found moderate correlations between suicidal ideation and the mental health and functioning scales (range: *r* = 0.28–0.62). The correlations between the independent item about difficulty doing usual activities at home/work and the mental health scales was moderate-to-high (range: *r* = 0.61–0.76), except alcohol (*r* = 0.08). For the short scales resulting from the IRT analysis, we found a similar pattern of correlations between suicide ideation and the shortened mental health scales as well as between the independent item and the mental health scales, except for alcohol use, for which we noted a substantial improvement (*r* = 0.34).

### Criterion validity

Table 4 presents the SCID diagnostic results. The majority of our sample (*n* = 85; 57%) met the modified SCID diagnostic criteria for at least one disorder: Major Depressive Disorder (21%), Post-traumatic Stress Disorder (47%), Generalized Anxiety Disorder (1%), Adjustment Disorder (7%), Alcohol Abuse (7%), and Alcohol Dependence (4%). In general, comorbidities were low, except for comorbidity of MDD and PTSD (14%).

**Table 4:** Group differences on MHAI scale scores by SCID diagnosis

	N	Score on Associated MHAI Sub-scale^a^	test statistic (df)^b^
SCID Diagnoses^c^			
Major Depression			
Diagnosis +	32	1.34 (0.08)	5.49 (48)***
Diagnosis -	118	0.82 (0.04)	
Post-traumatic Stress Disorder			
Diagnosis +	70	1.11 (0.06)	4.25 (143)***
Diagnosis -	80	0.78 (0.05)	
Alcohol Abuse			
Diagnosis +	10	25.9 (2.57)	7.46 (10)***
Diagnosis -	140	6.14 (0.64)	
Alcohol Dependence			
Diagnosis +	6	27.3 (2.97)	6.75 (5)***
Diagnosis -	144	6.62 (0.67)	

*** *p* < .001

^a^Associated MHAI scales for SCID diagnoses are as follows: MDD (IDSS); PTSD (GPTSS); Alcohol Abuse and Dependence (ASSIST 3.0)

^b^Unequal variance t-tests and the associated adjusted degrees of freedom

^c^+ sign indicates a positive diagnosis; − sign indicates a negative diagnosis

Concurrent criterion validity was assessed by comparing scale scores between SCID diagnosed cases and non-cases for MDD, PTSD, Alcohol Abuse, Alcohol Dependence. Group difference tests (Table 4) indicated highly significant differences comparing cases to non-cases: MDD (M = 1.34, SD = 0.08 vs. M = 0.82, SD = 0.04, *t* = 5.49, df(48), *p* < .001), PTSD (M = 1.11, SD = 0.05 vs. M = 0.78, SD = 0.06, *t* = 4.25, df(143), *p* < .001), alcohol abuse (M = 25.9, SD = 2.57 vs. M = 6.14, SD = 0.64, *t* = 7.46, df(10), p < .001), and alcohol dependence (M = 27.3, SD = 2.97 vs. M = 6.62, SD = 0.67, *t* = 6.75, df(5), *p* < .001). The distribution of scale scores for depression and post-traumatic stress by the associated diagnoses on the SCID are shown in Additional file 1: Appendix B.

Table 5 presents empirical cut-points, based on the Liu method of maximizing sensitivity, and test characteristics for the long and short versions of the MHAI scales. AUC statistics indicated sufficient or good differentiation for each of the four disorders. Overall accuracy estimates suggested fair-to-good average percentages of accurate classification by a given scale.

**Table 5:** Empirical cut-points and test characteristics of the long vs. short versions of the MHAI scales

	AUC statistic (SE) [95% CI]	Empirical cut-point (SE) [95% CI]	Se^b^	Sp^c^	Overall accuracy
Scale^a^					
Post-traumatic stress					
Long	0.66 (0.04) [0.59, 0.73]	0.915 (0.08) [0.75, 1.08]	0.66	0.66	0.66
Short	0.68 (0.04) [0.60, 0.75]	1.042 (0.07) [0.91, 1.17]	0.64	0.71	0.68
Depression					
Long	0.75 (0.03) [0.68, 0.81]	0.960 (0.12) [0.72, 1.20]	0.84	0.65	0.69
Short	0.78 (0.04) [0.71, 0.86]	1.065 (0.11) [0.84, 1.28]	0.81	0.75	0.76
Alcohol abuse					
Long	0.89 (0.03) [0.83, 0.95]	9.500 (6.27) [−2.78, 21.78]	1.00	0.78	0.80
Short	0.87 (0.05) [0.77, 0.96]	7.500 (0.74) [6.06, 8.94]	0.90	0.84	0.84
Alcohol dependence					
Long	0.93 (0.02) [0.88, 0.97]	14.500 (4.72) [5.24, 23.76]	1.00	0.86	0.87
Short	0.91 (0.03) [0.86, 0.96]	7.500 (0.93) [5.66, 9.33]	1.00	0.82	0.83

^a^Anxiety not included due to too few participants meeting SCID diagnostic criteria for anxiety disorders

^b^Se = sensitivity

^c^Sp = specificity

### Instrument refinement using item response theory

Based on our item inclusion criteria, we selected 8 MHAI items for depression, 12 for PTS (5 overlap with depression items, 1 with anxiety, and 6 are unique to PTS), 4 for anxiety, and 8 for impaired function for our shortened instrument. Discrimination parameters for the depression items ranged from *a =* 1.5 for the item “feeling tired, low in energy or slowed down” to *a =* 3.1 for the item “feeling sad.” Difficulty parameters ranged from *b*_1_ = − 1.4 for the item “feeling tired or fatigued” to *b*_3_ = 3.7 for the item “psychomotor agitation or slowing.” For the post-traumatic stress items, discrimination parameters ranged from *a =* 1.7 for “avoiding thoughts or memories of the event” to *a =* 3.0 “feeling that no one understands.” Location parameters ranged from *b*_1_ *=* − 1.5 for “feeling upset when reminded of the traumatic event,” to *b*_3_ *=* 2.9 for “trembling or shaking.” For the anxiety items, discrimination parameters ranged from *a =* 2.14 for “trembling or shaking” to *a =* 2.9 for “feeling tense or keyed up,” with location parameters ranging from *b*_1_ *=* − 1.0 for “feeling tense or keyed up” to *b*_3_ *=* 3.3 for “trembling or shaking.” Finally, for the functioning items, selected from the WHODAS and local function scales, discrimination parameters ranged from *a =* 1.9 “doing hobbies” to *a =* 3.2 for “conversing with others;” location parameters ranged from *b*_1_ *=* − 0.2 for “doing hobbies” to *b*_3_ *=* 3.10 for “helping others.”

Test information curves of the shorter scales indicated sufficient and comparable reliability across the latent trait spectrum compared to the longer scales. Validity and reliability results for the shortened scales were comparable to the longer scores. The IRT approach yielded comparable or slightly higher AUCs for the shorter scales compared to the longer ones, indicating that selecting fewer, but high performing, items tended to increase diagnostic accuracy.

## Discussion

This paper described the adaptation and psychometric testing of a set of scales of mental health and alcohol use problems in a sample of approximately 150 conflict-affected Ukrainians, namely IDPs and military veterans. Using a systematic approach, including the incorporation of locally relevant items based on a prior qualitative study we conducted in the same population, we generated a brief, reliable, and valid measure of three mental health problems (depression, post-traumatic stress, and anxiety) and alcohol use problems. The measure, which for convenience we refer to as the Mental Health Assessment Inventory (MHAI), can be used among male and female conflict-affected adults in Ukraine.

Psychometric testing entailed evaluation of internal consistency reliability and test-retest reliability as well as both convergent construct validity and concurrent criterion validity. Criterion validity was evaluated through the use of a standardized clinical diagnostic tool, the Structured Clinical Interview for DSM-IV-Research Version (SCID). We created a more pragmatic yet psychometrically robust version of the valid measure based on Item Response Theory (IRT) analyses. These analyses identified key symptoms and function items that, taken together, increased our diagnostic accuracy while shortening the time it takes to complete the assessment.

Approximately half of the participants met diagnostic criteria for Major Depressive Disorder, Post-traumatic Stress Disorder, Alcohol Abuse, and/or Alcohol Dependence. In comparing SCID-defined cases to non-cases for each disorder, we found significant differences (*p* < .001) on each of the scale scores for depression, post-traumatic stress, and alcohol use problems, providing evidence of concurrent validity for the corresponding scales in the MHAI. The empirical estimates of diagnostic accuracy for the MHAI scales provided some additional evidence of their validity. Diagnostic accuracy was moderate for the post-traumatic stress scale and fairly strong for the depression and alcohol use scales. The short scales can be used and still achieve the same (or better) classification accuracy as the long scales. The empirical cut-points we used maximized sensitivity and specificity. Given the lack of psychometric research from the region with which to compare our findings, these results need to be interpreted with caution. Modifications to the cut-off score, such as by lowering it, may be appropriate if screening high-need individuals into mental health services is the ultimate goal, as connecting such individuals to care may counterbalance a higher false-positive rate. We echo others in highlighting the need for more research to calibrate screening instruments like these in studies of mental health in conflict-affected populations [44].

Regarding reliability, overall we found very good estimates of internal consistency and test-retest reliability in our measures for symptoms of depression, post-traumatic stress, anxiety, and alcohol use. Cronbach’s alpha values for internal consistency reliability were consistently above 0.80, and the IRT-based analyses revealed the shortened versions of these scales were comparable to or, in some cases, better than the full versions of the scales in the MHAI. The coefficients for test-retest reliability were also consistently above 0.80, and we found comparable results in the IRT-based analyses, suggesting either the short or long versions of the scale can produce consistent results.

IRT analysis is an alternative—as opposed to a substitute—to standard reliability and validity analyses based on classical test theory (CTT). We elected to use IRT, in addition to CTT, because it can describe more finely the error typical of individual scale items written to tap into unobservable constructs, such as depression, post-traumatic stress, and anxiety [11]. In psychiatric research, it is becoming increasingly recognized that IRT can assist instrument developers to identify particular scale items that best discriminate among individuals with regard to the level of intensity they experience the latent construct (e.g., depression) [45]. This recognition has extended to mental health research on conflict-affected populations. For example, Betancourt and colleagues used IRT to refine a dimensional scale of psychosocial adjustment in Ugandan youth living in IDP camps [46], and Haroz and colleagues used IRT to compare the performance of the Hopkins Symptom Checklist 15-item (HSCL-15) depression scale across eight countries [47].

Surprisingly, we did not find high correlations between the mental health symptom scales and alcohol use or between the functioning scales and alcohol use. This is in contrast to other studies of displaced and veteran populations, both within and outside the region, which have found alcohol use to be highly correlated with mental health problems and functioning [48–50]. It is possible the scale for assessing alcohol use problems (ASSIST 3.0) was not sufficiently sensitive to differentiate problematic from non-problematic use.

Although we found good evidence of concurrent criterion validity comparing SCID-defined cases to non-cases on the MHAI sub-scales for depression, post-traumatic stress, and alcohol use, very few participants met the diagnostic criteria for anxiety disorder, so we were not able to assess criterion validity for the anxiety scale of the MHAI. The reason for few anxiety diagnoses may have resulted from our sampling strategy, whereby we purposefully asked recruiters to refer people based on presentation of symptoms related to depression, post-traumatic stress, and alcohol misuse. Alternatively, the SCID criteria for PTSD or Adjustment Disorder may have better accounted for the symptomatology in this population than generalized anxiety. Cases of anxiety may have, therefore, been captured in other diagnostic categories prioritized in the SCID assessment. We note the SCID has not been widely used in eastern Europe, and we found no prior research testing its use in Ukraine. Notably, the SCID-5, which corresponds to the latest DSM criteria, has not yet been translated into a Ukrainian or Russian language version; we acknowledge the use of its predecessor as a study limitation.

This study had several other limitations. We sampled adult individuals from only two urban areas, although there are veterans and displaced individuals and families scattered across the country. The study sample size is relatively small, although it is similar to those of other psychometric studies conducted by our group [24, 51] as well as other groups [37, 52] in different populations in low- and middle-income countries. We also note that our a priori sample size calculation indicated that 45 participants in each group under study provided sufficient power to detect medium differences on symptom scores between the groups.

Our study was strengthened by working in partnership with local mental health experts, and due to the availability of a mental health workforce in Ukraine we were able to employ Ukrainian mental health professionals to use a standardized diagnostic tool (SCID) for evaluating the validity of the MHAI. We took care to ensure the quantitative assessments reflected the findings of our prior qualitative research involving IDPs, veterans of the conflict, non-IDP Ukrainian citizens, and mental health care workers. While much research on conflict-affected populations (pertinently) focuses on symptoms of depression and trauma [53, 54], we also attended to alcohol use.

## Conclusion

Accurate mental health research and appropriate service delivery requires reliable, valid, and useful measurement tools. The literature repeatedly calls attention to the high need for validated measures for both epidemiologic and clinical purposes. These kinds of measures are frequently lacking for conflict-affected populations, owing to difficulty and cost of local adaptation and testing. The methods and procedures used in this study (and based on research described elsewhere [10, 11, 13]) were designed for relatively rapid investigations among conflict-affected populations.

To our knowledge, this is the first validity study of instruments to assess for mental health and alcohol use problems among Ukrainians affected by the current conflict. The resulting instrument is being used to facilitate enrollment screening and symptom tracking in a psychotherapeutic intervention for adult Ukrainian IDPs, veterans, and family members of veterans and will also be made freely available to other researchers and clinical workers in Ukraine. This study also demonstrated how IRT can produce shortened versions of measures that retain comparable—and, in some instances, improved—psychometric properties compared with the longer versions. We suggest that measurement methods based on IRT, in addition to those based on classical test theory, should become a standard practice in validity studies of common psychiatric and behavioral conditions.

## Additional file

Additional file 1: **Appendix A**. Mental Health Assessment Inventory (MHAI) scales for depression, post-traumatic stress, anxiety, alcohol use, and functioning. **Appendix B**. Distributions of scale scores for depression and post-traumatic stress by associated diagnoses on the SCID. (DOCX 144 kb)

## Abbreviations

ASSIST: Alcohol Smoking and Substance Involvement Screening Test
AUC: Area under the curve
DIME: Design, Implementation, Monitoring, and Evaluation
DSM: Diagnostic and Statistical Manual of Mental Disorders
GAD: Generalized Anxiety Disorder
GPTSS: Global Post-traumatic Stress Scale
GRM: Graded response model
IDP: Internally Displaced Person
IDSS: International Depression Symptom Scale
IRT: Item Response Theory
MDD: Major Depressive Disorder
MHAI: Mental Health Assessment Inventory
PCA: Principle components analysis
PTS: Post-traumatic stress
PTSD: Post-traumatic Stress Disorder
RCT: Randomized controlled trial
SCID: Structured Clinical Interview for DSM
SCID-IV-RV: Structured Clinical Interview for DSM IV – Revised Version
TIC: Test information curve
WHO: World Health Organization
WHODAS: World Health Organization Disability Adjustment Schedule
