# Supplementary material for: Adaptation and testing of an assessment for mental health and alcohol use problems among conflict-affected adults in Ukraine
Source: Confl Health. 2018 Aug 15;12:34. doi: 10.1186/s13031-018-0169-6 (PMC6092824; doi:10.1186/s13031-018-0169-6)
Supplement: Supplementary file 1 — Appendix A. Mental Health Assessment Inventory (MHAI) scales for depression, post-traumatic stress, anxiety, alcohol use, and functioning. Appendix B. Distributions of scale scores for depression and post-traumatic stress by associated diagnoses on the SCID. (DOCX 144 kb) [file 13031_2018_169_MOESM1_ESM.docx]

**APPENDIX A.** Mental Health Assessment Inventory (MHAI) scales for depression, post-traumatic stress, anxiety, alcohol use, and functioning.

| **Appendix A. Items used on MHAI sub-scales for depression, anxiety, post-traumatic stress, and local functioning.** | | | | | | | | |
| --- | --- | --- | --- | --- | --- | --- | --- | --- |
| **Item No.** | **Source** | | | | | | **MHAI Sub-Scale** | **Item** |
|  | **QD** | **PL** | **HTQ** | **HSCL** | **DSM** | **A3.0** |  |  |
| 1 | ● | ● |  |  |  |  | PTS | Feeling afraid/fearful |
| 2 |  | ● |  |  |  |  | PTS | Feeling isolated |
| 3 | ● | ● | ● |  | ● |  | PTS | Feeling detached from others |
| 4 |  | ● | ● |  |  |  | PTS | Unable to experience positive emotions |
| 5 |  | ● | ● |  | ● |  | PTS^*^ | Repeated or disturbing memories, thoughts or images about the trauma |
| 6 | ● | ● | ● |  | ● |  | PTS | Repeated trauma-related dreams/nightmares |
| 7 |  | ● |  |  |  |  | PTS | Feeling a sense of loss |
| 8 |  | ● |  |  |  |  | PTS | Intimate relationship problems |
| 9 |  | ● |  |  | ● |  | PTS | Reckless or self-destructive behavior |
| 10 |  | ● | ● |  |  |  | PTS | Feeling agitated |
| 11 | ● | ● | ● |  | ● |  | PTS^*^ | Feeling emotionally upset when something reminded you of the stressful event |
| 12 | ● | ● | ● |  |  |  | PTS^*^ | Feeling that no one understands |
| 13 |  | ● |  |  |  |  | PTS | Fights or difficulty getting along with my family |
| 14 |  | ● |  |  |  |  | PTS | Feeling unable to cope |
| 15 | ● | ● | ● |  | ● |  | PTS | Angry outbursts |
| 16 | ● | ● | ● |  | ● |  | PTS | Feeling jumpy or easily startled |
| 17 |  | ● | ● |  |  |  | PTS | Feeling guilty |
| 18 |  | ● |  |  |  |  | PTS | Unable to socialize with others |
| 19 |  |  |  |  | ● |  | PTS | Feeling super alert, watchful, or on guard |
| 20 |  | ● | ● |  | ● |  | PTS | Acting or feeling as if the past stressful event is happening again, as if you were reliving it |
| 21 |  | ● | ● |  | ● |  | PTS | A physical reaction when reminded of the past stressful event (for example: heart racing, sweating, shaking, rapid breathing, feeling faint or nauseous) |
| 22 |  | ● |  |  | ● |  | PTS^*^ | Avoiding thoughts/memories related to the past stressful event |
| 23 |  | ● | ● |  | ● |  | PTS^*^ | Avoiding activities or situations that remind you of the past stressful event |
| 24 |  | ● | ● |  |  |  | PTS | Unable to remember parts of the past stressful event |
| 25 |  | ● | ● |  | ● |  | PTS | Blaming yourself or others for what happened |
| 26 |  | ● |  |  |  |  | PTS^*^ | Feeling a loss of your sense of self |
| 27 |  | ● | ● |  |  |  | PTS | Feeling that I have no one to rely on |
| 28 |  | ● |  |  |  |  | PTS | Feeling a loss of faith |
| 29 |  | ● |  |  |  |  | PTS | Feeling that your surroundings or the environment around you are not real |
| 30 |  | ● |  |  |  |  | PTS | Frequently trembling or shaking |
| 31 |  | ● |  |  |  |  | PTS | Feeling of indifference to what is happening around |
| 32 |  | ● |  |  |  |  | PTS | Not being able to remember things or being forgetful |
| 1 |  | ● |  | ● |  |  | Depression, PTS^*#^ | Feeling sad |
| 2 | ● | ● |  | ● |  |  | Depression, PTS^*#^ | Feeling no interest in things/less interest in daily activities |
| 3 |  | ● |  | ● |  |  | Depression, PTS^*#^ | Crying easily |
| 4 | ● | ● |  | ● |  |  | Depression, PTS | Feeling hopeless about the future |
| 5 | ● | ● |  | ● |  |  | Depression, PTS^*#^ | Feeling lonely; Feeling isolated |
| 6 | ● |  |  |  |  |  | Depression | Avoiding others |
| 7 |  | ● |  | ● |  |  | Depression, PTS^*#^ | Feeling tired, low in energy or slowed down |
| 8 |  | ● |  |  |  |  | Depression, PTS | Weighing too little |
| 9 |  | ● |  |  |  |  | Depression | Weighing too much |
| 10 |  | ● |  | ● |  |  | Depression, PTS | Problems with my appetite |
| 11 | ● | ● |  | ● |  |  | Depression, PTS | Problems with my sleep; disturbed sleep |
| 12 |  |  |  | ● |  |  | Depression**^#^** | Feeling of being trapped or caught |
| 13 |  | ● |  | ● |  |  | Depression, PTS | Worrying too much about things |
| 14 |  | ● |  | ● |  |  | Depression**^#^** | Feelings of worthlessness |
| 15 |  | ● |  |  |  |  | Depression, Anxiety | Headache |
| 16 |  | ● |  |  |  |  | Depression | Stomach aches |
| 17 |  | ● |  |  |  |  | Depression, PTS | Other bodily aches and pains |
| 18 |  | ● |  |  |  |  | Depression | Feeling Angry |
| 19 |  | ● |  |  |  |  | Depression, PTS | Thinking too much |
| 20 |  | ● |  |  |  |  | Depression | Feeling confused |
| 21 |  | ● |  |  |  |  | Depression | Feeling weakness in your heart |
| 22 |  | ● |  |  |  |  | Depression | Heart palpitations |
| 23 |  | ● |  |  |  |  | Depression | Feeling as though your heart or chest is heavy |
| 24 |  | ● |  |  |  |  | Depression, PTS | Feeling pressure on your heart or chest |
| 25 |  | ● |  |  |  |  | Depression, PTS | Pain in your heart or chest |
| 26 |  |  |  |  | ● |  | Depression**^#^** | Moving or speaking so slowly or so fast that others have noticed |
| 27 |  |  |  |  | ● |  | Depression, PTS | Difficulty concentrating |
| 28 |  | ● |  |  |  |  | Depression, PTS | Difficulty doing your usual activities at home or work |
| 29 | ● | ● |  | ● |  |  | Depression, PTS | Thoughts of wanting to kill yourself; propensity for suicide |
| 1 |  |  |  | ● |  |  | Anxiety | Suddenly scared for no reason |
| 2 | ● |  |  | ● |  |  | Anxiety | Feeling fearful |
| 3 |  |  |  | ● |  |  | Anxiety | Faintness, dizziness, or weakness |
| 4 | ● |  |  | ● |  |  | Anxiety**^^^** | Nervousness or shakiness inside |
| 5 |  |  |  | ● |  |  | Anxiety | Heart pounding or racing |
| 6 |  |  |  | ● |  |  | Anxiety**^^^**^*^ | Trembling |
| 7 | ● |  |  | ● |  |  | Anxiety**^^^** | Feeling tense or keyed up |
| 8 |  |  |  | ● |  |  | Anxiety**^^^** | Spells of terror or panic |
| 9 |  |  |  | ● |  |  | Anxiety | Feeling restless, can’t sit still |
| 1 | ● |  |  |  |  |  | Local Functioning | Taking care of your personal appearance |
| 2 | ● |  |  |  |  |  | Local Functioning | Taking care of your health |
| 3 | ● |  |  |  |  |  | Local Functioning | Spending time with your family |
| 4 | ● |  |  |  |  |  | Local Functioning | Becoming part of the community |
| 5 | ● |  |  |  |  |  | Local Functioning | Taking care of children |
| 6 | ● |  |  |  |  |  | Local Functioning | Earning money and looking for employment |
| 7 | ● |  |  |  |  |  | Local Functioning | Helping your family |
| 8 | ● |  |  |  |  |  | Local Functioning | Doing household activities (for example: cooking, cleaning, washing) |
| 9 | ● |  |  |  |  |  | Local Functioning | Joining in community activities and cultural events (for example: going on walks, going to museums, going to the theater) |
| 10 | ● |  |  |  |  |  | Local Functioning | Learning to live in a new community |
| 11 | ● |  |  |  |  |  | Local Functioning | Trying to live in given conditions |
| 12 | ● |  |  |  |  |  | Local Functioning | Spending time with people like you |
| 13 | ● |  |  |  |  |  | Local Functioning | Looking for services and benefits / registration of benefits |
| 14 | ● |  |  |  |  |  | Local Functioning | Receiving education / attending trainings |
| 15 | ● |  |  |  |  |  | Local Functioning | Helping others |
| 16 | ● |  |  |  |  |  | Local Functioning | Playing sports / exercise |
| 17 | ● |  |  |  |  |  | Local Functioning | Doing hobbies (for example: making crafts, sewing, embroidery, hunting, fishing, reading, games) |
| 18 | ● |  |  |  |  |  | Local Functioning | Conversing with others |
| 19 | ● |  |  |  |  |  | Local Functioning | Spending time in nature (for example: working the land, walking outdoors) |
| 20 | ● |  |  |  |  |  | Local Functioning | Making / keeping friends |
| 21 | ● |  |  |  |  |  | Local Functioning | Creating conditions for comfortable living |
| 1 |  |  |  |  |  | ● | Alcohol use | In your life, have you ever used alcoholic beverages (beer, wine, spirits, etc.)? [Subsequent questions only asked if answer to this question is affirmative.] |
| 2 |  |  |  |  |  | ● | Alcohol use**`** | In the past three months, how often have you used alcoholic beverages? |
| 3 |  |  |  |  |  | ● | Alcohol use**`** | During the past three months, how often have you had a strong desire or urge to use alcoholic beverages? |
| 4 |  |  |  |  |  | ● | Alcohol use | During the past three months, how often has your use of alcoholic beverages led to health, social, legal, or financial problems? |
| 5 |  |  |  |  |  | ● | Alcohol use | During the past three months, how often have you failed to do what was normally expected of you because of your use of alcoholic beverages? |
| 6 |  |  |  |  |  | ● | Alcohol use | Has a friend or relative or anyone else ever expressed concern about your use of alcoholic beverages? |
| 7 |  |  |  |  |  | ● | Alcohol use | Have you ever tried and failed to control, cut down, or stop using alcoholic beverages? |
| Sources: QD = qualitative data; PL = published literature; HTQ = Harvard Trauma Questionnaire; HSCL = Hopkins Symptom Checklist-25; DSM = Diagnostic and Statistical Manual of Mental Disorders; A3.0 = ASSIST 3.0 | | | | | | | | |
| Symbols indicating inclusion in short versions of scales: ***** pts, **^#^** depression, **^** anxiety, **`** alcohol. | | | | | | | | |

**APPENDIX B. Distributions of scale scores for depression and post-traumatic stress by associated diagnoses on the SCID.**
